# Supplementary material for: Antibody-directed extracellular proximity biotinylation reveals that Contactin-1 regulates axo-axonic innervation of axon initial segments
Source: Nat Commun. 2023 Oct 26;14:6797. doi: 10.1038/s41467-023-42273-8 (PMC10603070; doi:10.1038/s41467-023-42273-8)
Supplement: Supplementary file 1 — Supplementary Information [file 41467_2023_42273_MOESM1_ESM.pdf]

SUPPLEMENTARY INFORMATION

Antibody-directed extracellular proximity biotinylation reveals Contactin-1 regulates axo-axonic innervation of axon initial segments.

Ogawa and Lim *et al.*

Supplementary information includes:

Supplementary Figures S1-S8

Supplementary extended materials, detailed listing of all reagents, plasmids, antibodies, sources, etc.

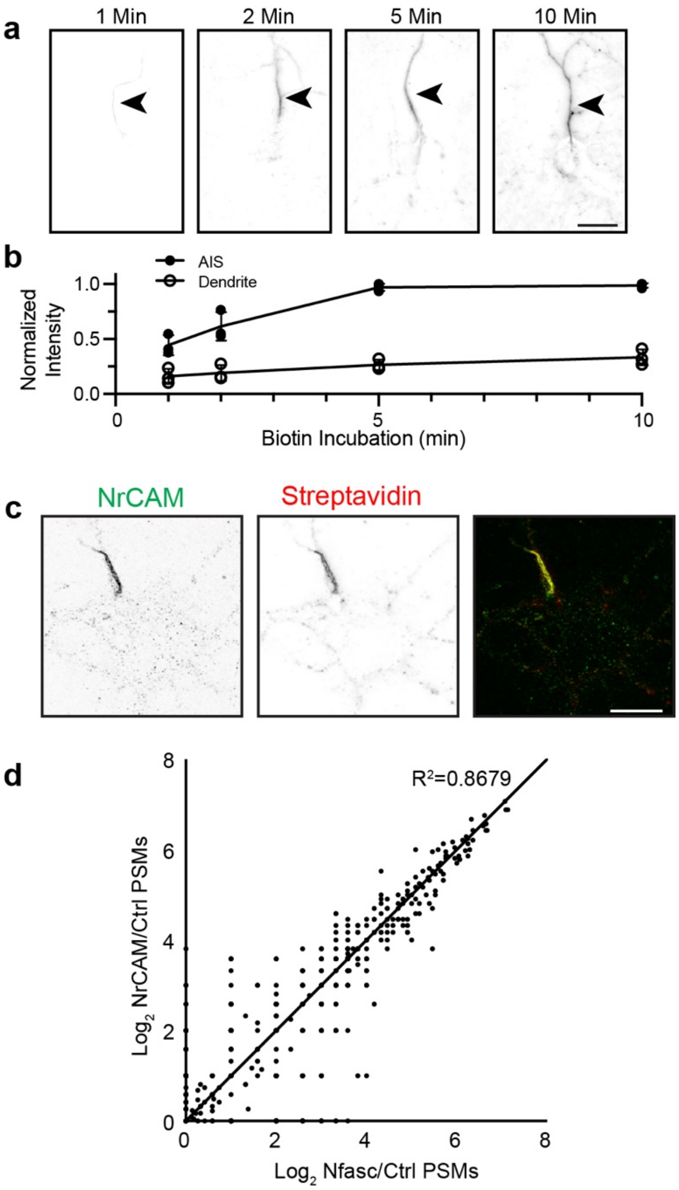

Figure S1. BAR-Nfasc and BAR-NrCAM are highly concordant. a, b, Streptavidin labeling (i.e. biotinylation) of the AIS (arrowhead) as a function of biotin-tyramide incubation time. N = 3 independent experiments. Error bars, ±SEM. Scale bar, 20 μm. **c**, Fluorescence imaging of DIV14 rat hippocampal neurons labeled by NrCAM-BAR. NrCAM immunofluorescence (green) enrichment defines the AIS. Biotinylated proteins were detected using Alexa594-conjugated streptavidin. Scale bar, 20 μm. **d**, Scatter plot of the number of peptide spectral matches

(PSMs) for each biotinylated protein identified by mass spectrometry using either Nfasc-BAR or NrCAM-BAR. Data were highly concordant since most proteins identified fell on or close to the solid line representing equal enrichment in both BAR conditions. N=1 experiment.

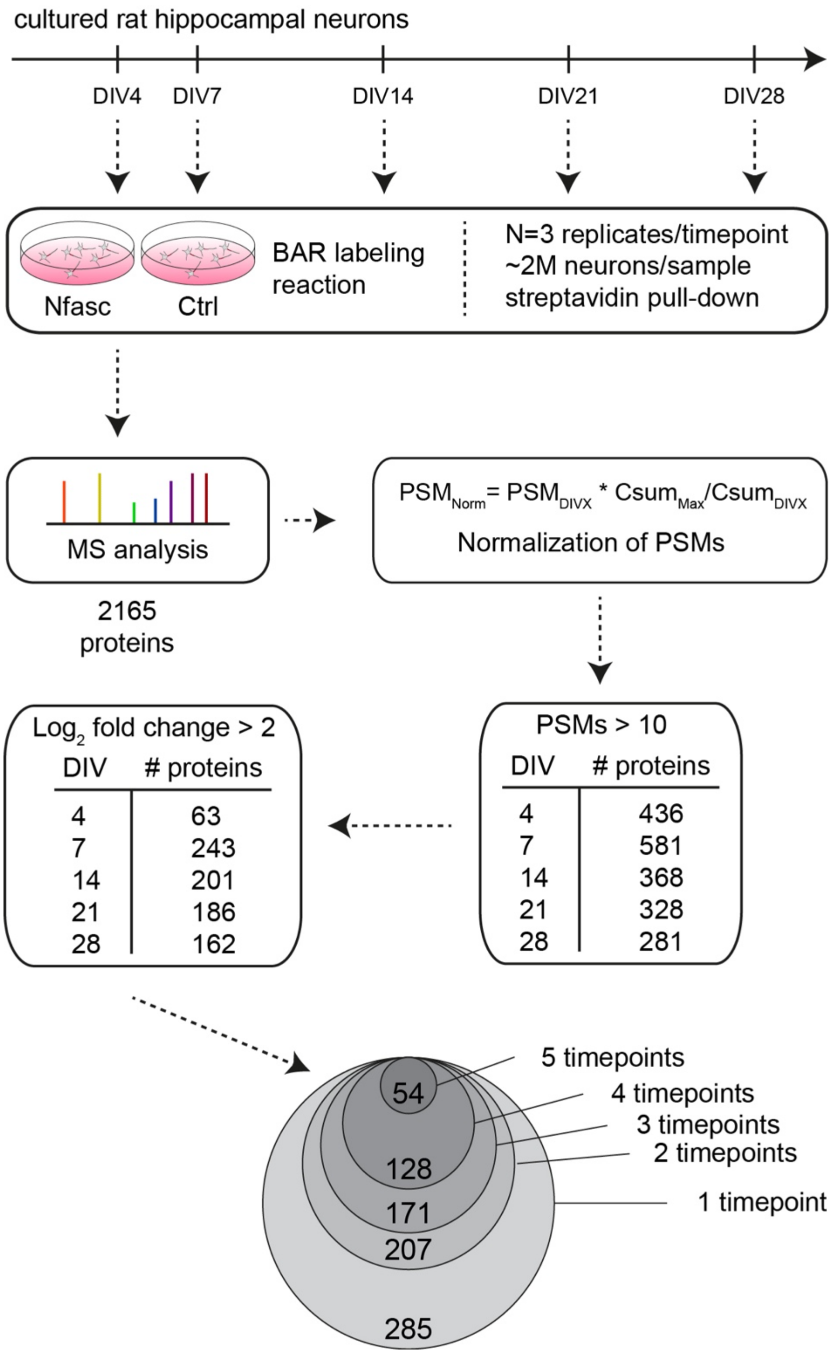

Figure S2. Workflow of Nfasc-BAR labeling and normalization of PSMs. Nfasc-BAR labeling was performed three times independently for each developmental timepoint with 2 million neurons per experiment. Mass spectrometry analysis resulted in a dataset of 2165 proteins. To compare across timepoints, PSMs were normalized using the formula $PSM_{i,}^{Norm,DivX} = PSM_{i,}^{DivX}$ *

$SPSM_{Carboxylases}^{DivY}$ / $SPSM_{Carboxylases}^{DivX}$, where i is a particular protein on the set, X is a Div time point, Y is the Div time point with the maximum value for summed carboxylase PSMs. Candidates were distinguished from background by filtering based on the number of PSMs identified and the fold change. Supplementary data files show the number of proteins that satisfied one or both criteria at a given timepoint. Circles show the number of proteins that satisfied both criteria for 1-5 timepoints.

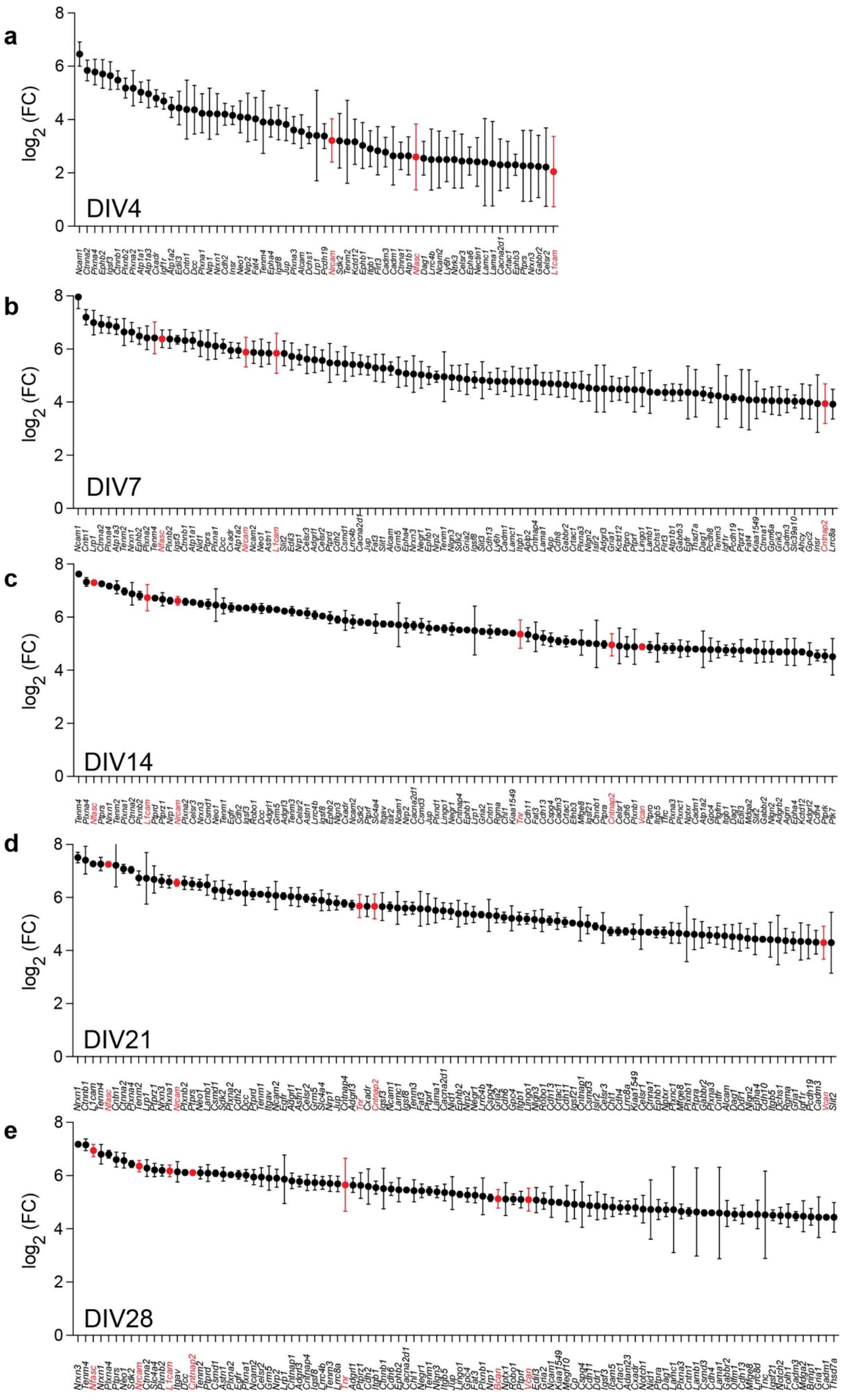
a
log2 (FC)
DIV4
b
log2 (FC)
DIV7
c
log2 (FC)
DIV14
d
log2 (FC)
DIV21
e
log2 (FC)
DIV28

Figure S3. Fold-enrichment for the top 100 most enriched proteins identified using Nfasc-BAR during *in vitro* neuron development. a-e, Log_2 fold-change (log_2(FC)) for the top 100 most enriched proteins identified at DIV 4 (**a**), 7 (**b**), 14 (**c**), 21 (**d**) and 28 (**e**). Known AIS proteins are indicated in red. Error bars, ±SEM. N=3 for each time point.

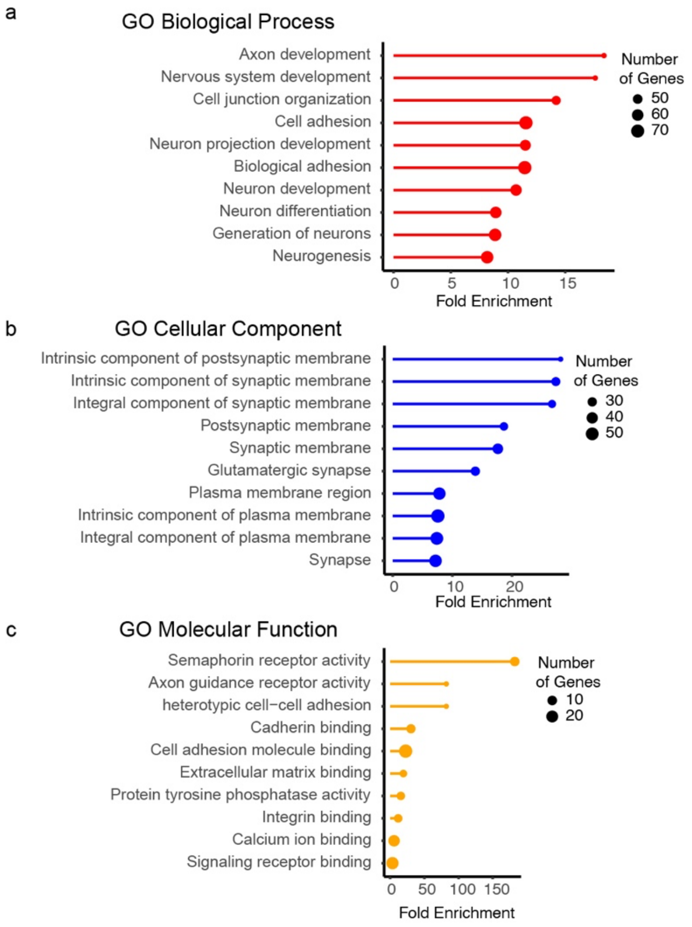

Figure S4. Gene Ontology analysis at DIV14. The top 100 genes by fold-enrichment analyzed using Biological Process (a), Cellular Component (b), and Molecular Function (c) pathway databases. Gene Ontology analysis was performed using ShinyGO v0.77.

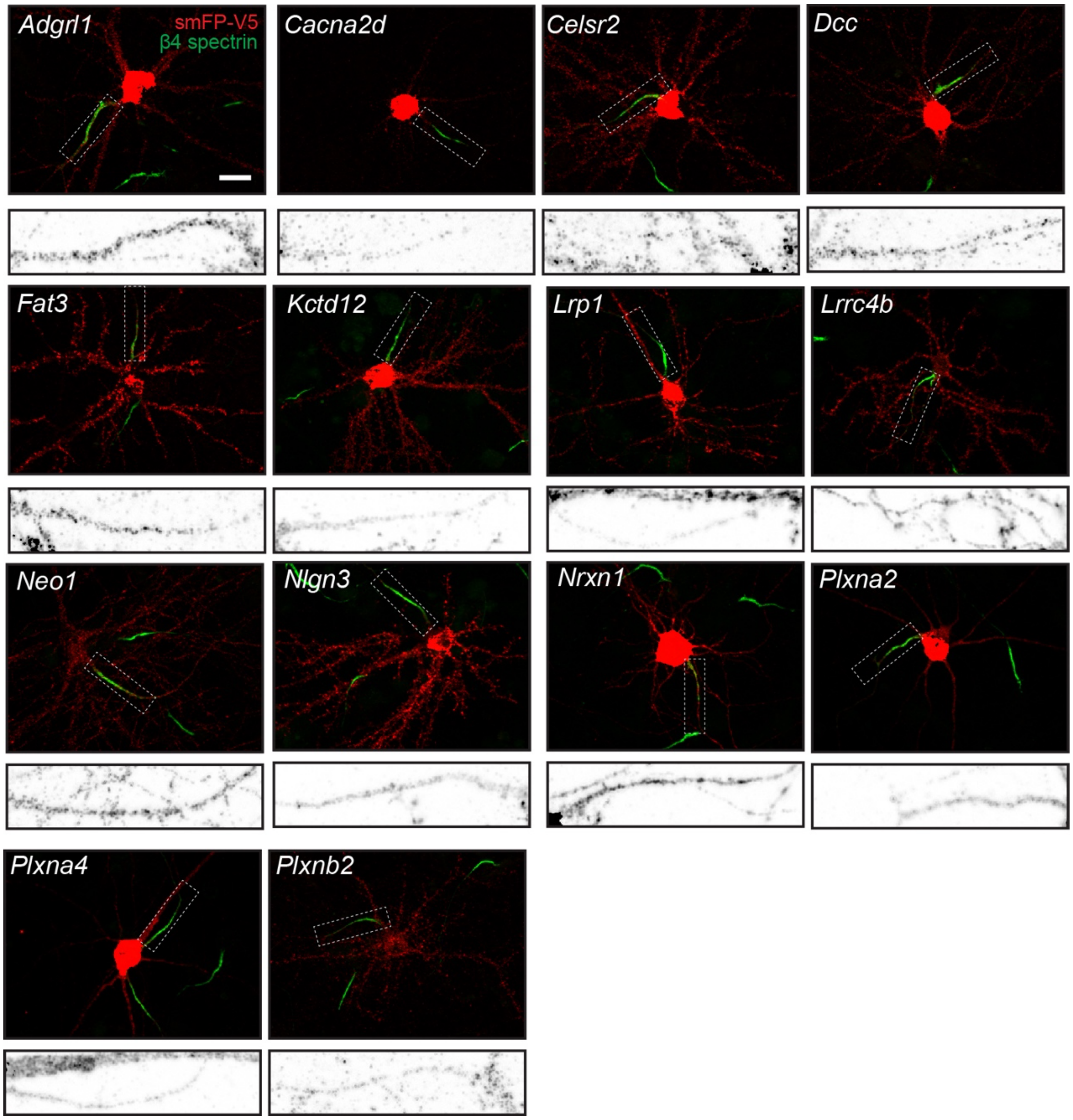

Figure S5. Tagging of endogenous membrane proteins. Examples of smFP-V5 tagged proteins (red). AIS are labeled for β4 spectrin (green). The inset boxed regions correspond to the tagged protein at the AIS. Neurons were transfected at DIV0 and fixed at DIV21. N=2 independent experiments with two independent viruses targeting each gene of interest. Scale bar, 20 µm.

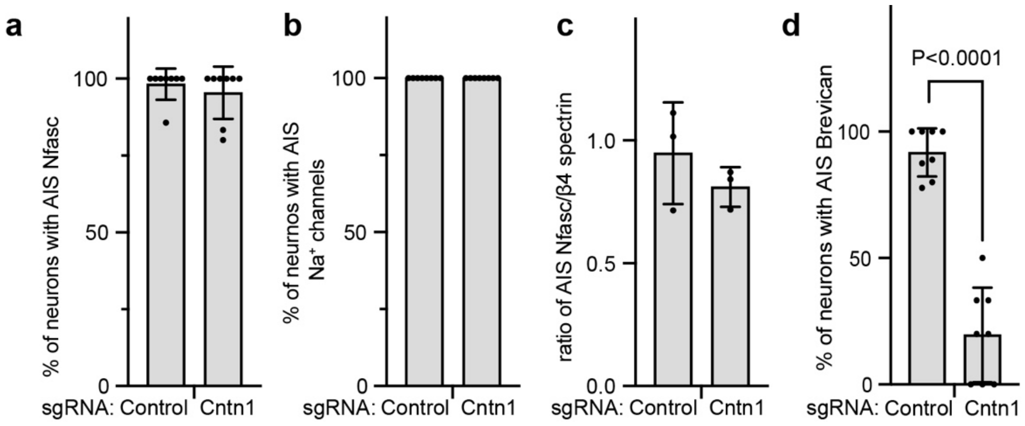

Figure S6. Loss of AIS Cntn1 has no effect on AIS structure or position, but is essential for Brevican recruitment. a, b, The percentage of neurons with AIS Nfasc (**a**) or Na^+ channels (**b**) in control and Cntn1-deficient neurons. For AIS Nfasc, N=2 independent coverslips with 8 fields of view total; each dot represents a different field of view. n= 57 and 46 neurons, for control and Cntn1 sgRNA, respectively. Error bars, ±SEM, (t=0.798, df=14) p=0.4382. For AIS Na^+ channels, N=2 independent coverslips with 8 fields of view total; each dot represents a different field of view. n= 57 and 41 neurons, for control and Cntn1 sgRNA, respectively. All neurons analyzed in both conditions were labeled with AIS Na^+ channels. **c,** Loss of Cntn1 using CRISPR-mediated genome editing has no effect on the enrichment of AIS Nfasc. Quantification of the ratio of AIS Nfasc to β4 spectrin. N= 3 independent coverslips, with a total of 84 and 50 neurons for control and Cntn1 sgRNA, respectively. Error bars, ±SEM, Error bars, ±SEM, (t=0.1.072, df=4) p=0.3440. **d,** The percentage of neurons with AIS Brevican in control and Cntn1-deficient neurons. N= 2 independent coverslips with 8 fields of view, with a total of 52 and 49 neurons for control and Cntn1 sgRNA, respectively. Error bars, ±SEM, (t=9.724, df=14) $p=1.3X10^{-7}$. In all experiments neurons were transduced at DIV0 and analyzed at DIV21.

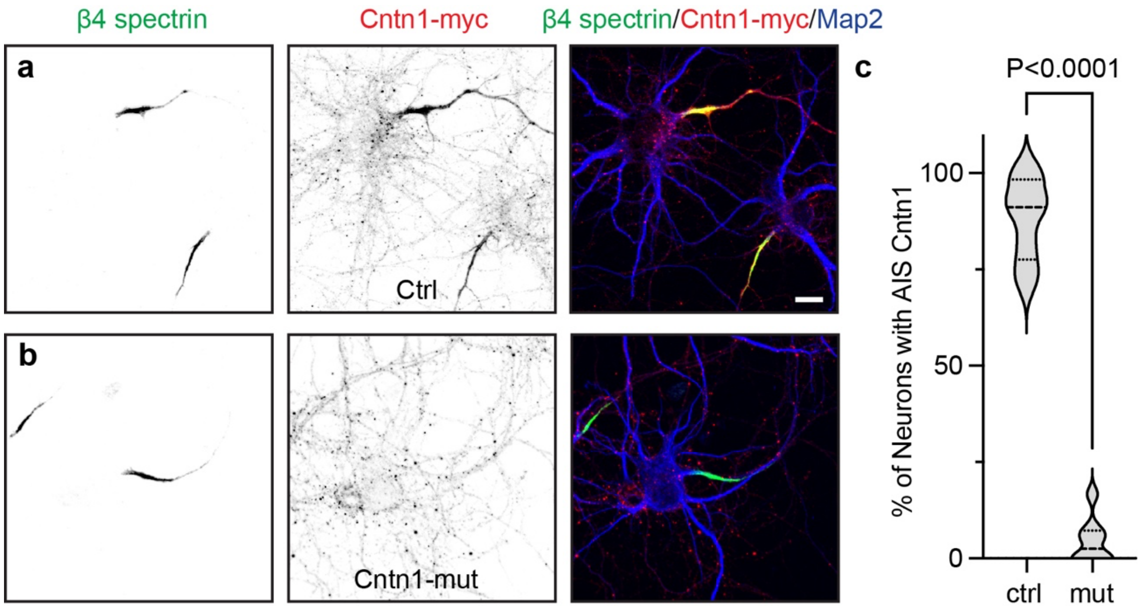

Figure S7. Cntn1 mutants that cannot bind Nfasc fail to cluster at the AIS. a, b, Immunostaining of neurons transfected at DIV0 and fixed at DIV10 using Cntn1-myc (**a**) or a myc-tagged Cntn1-mut (**b,** a mutant Cntn1 with three point mutations disrupting Nfasc interaction). AIS are labeled for β4 spectrin (green), myc (red) and Map2 (blue). Scale bar, 10 µm. **c,** The percentage of transduced neurons with Cntn1 enrichment at the AIS for Cntn1-myc or Cntn1-mut. N= 8 fields of view from 2 experiments; a total of 124 and 116 neurons transduced with Cntn1-myc or Cntn1-mut, respectively, was measured. In the violin plot the dashed lines indicate the median, while the dotted lines represent the first and third quartiles. ($t=19.53$, $df=14$) $p=1.5 \times 10^{-11}$.

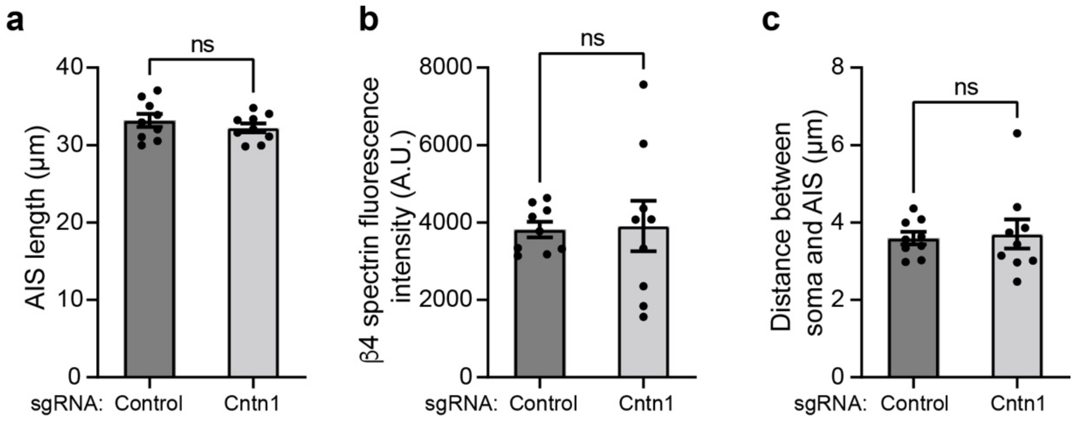

Figure S8. Cntn1 depletion in PyNs does not affect AIS structure or position. (**a**) Quantification of the average AIS length of HA+ PyNs in layer II/III of the somatosensory cortex from *Nkx2.1-creER;Ai9* mice co-electroporated at E15.5 with a plasmid expressing Cas9 and a plasmid expressing smFP-HA and a control sgRNA or Cntn1 sgRNA; mice were sacrificed at P17. β4-spectrin immunostaining was used for visualization and measurement of the AIS length. (t=0.9513, df=16) p=0.3556. (**b**) Quantification of the relative integrated fluorescence intensity of β4-spectrin at the AIS of HA+ PyNs in layer II/III of the somatosensory cortex from *Nkx2.1-creER;Ai9* mice co-electroporated at E15.5 with plasmids indicated in (**a**) and sacrificed at P17. (t=0.135, df=16) p=0.8943. (**c**) Quantification of the distance between the cell soma and the start of the AIS (defined by β4 spectrin immunolabeling) of HA+ PyNs in layer II/III of the somatosensory cortex from *Nkx2.1-creER;Ai9* mice co-electroporated at E15.5 with plasmids indicated in (**a**) and sacrificed at P17. (t=0.2600, df=16) p=0.7982. For (**a-c**), 5 AISs from 3 fields of view from 3 animals were analyzed for each condition (Student's t tests). NS = not significant. Data are mean ± SEM.

SUPPLEMENTARY MATERIALS AND REAGENTS

Reagents, antibodies, and plasmids used in this study are summarized below:

REAGENT or RESOURCE	SOURCE	IDENTIFIER
**Antibodies**		
Mouse anti-AnkG (IgG2a)	NeuroMab	Cat# 75-146; RRID: AB_10673030
Mouse anti-Gephyrin (IgG1)	Synaptic Systems	Cat# 147011; RRID: AB_887717
Guinea pig anti-VGAT	Synaptic Systems	Cat# 131004; RRID: AB_887873
Guinea pig anti-Brevican	Gift of Dr. Constanze Seidenbecher	N/A
Rabbit monoclonal anti-HA	Cell Signaling Technology	Cat# 3724; RRID: AB_1549585
Mouse anti-Myc	MBL International Corporation	Cat# M192 PRID: AB_11160947
Mouse anti-PSD95	Antibodies Incorporated	Cat# 75-028 PRID: AB_2292909
Mouse anti-Tenascin-R	R&D Systems	Cat# MAB1624 RRID: AB_2207001
Mouse anti-Tuj1	BioLegend	Cat# 801202 RRID: AB_10063408
Mouse anti-V5	Invitrogen	Cat# R960CUS RRID: AB_159298
Rabbit anti-βIV-Spectrin	Rasband lab	RRID: AB_2315634
Rabbit anti-Kv1.2	Gift of Dr. James Trimmer	RRID: 2756300
Rabbit anti-NrCAM	Abcam	Cat# ab24344 RRID: AB_448024
Chicken anti-MAP2	Encor	Cat# CPCA-MAP2
Chicken anti-Neurofascin	R&D Systems	Cat# AF3235 RRID: AB_10890736
Goat anti-Cntn1	R&D Systems	Cat# AF904 RRID: AB_2292070
Rat anti-HA	Millipore Sigma	Cat# 11867423001 RRID: AB_390918
HRP-conjugated goat anti-chicken IgY	Aves Labs	Cat# H-1004 RRID: AB_2313517
HRP-conjugated goat anti-rabbit IgG	Jackson ImmunoResearch Labs	Cat# 111-035-003 PRID: AB_2313567
Alexa Fluor 594 conjugated streptavidin	Thermo Fisher Scientific	Cat# S11227
Aminomethylcoumarin (AMCA) conjugated goat anti-chicken IgY	Jackson ImmunoResearch Labs	Cat# 103-155-155 RRID: AB_2337385
Aminomethylcoumarin (AMCA) conjugated goat anti-rat IgG	Thermo Fisher Scientific	Cat# A21093 PRID: AB_2535748
Alexa Fluor 488 conjugated goat anti-chicken IgY	Jackson ImmunoResearch Labs	Cat# 103-545-155 RRID: AB_2337390
Alexa Fluor 488 conjugated goat anti-mouse IgG	Thermo Fisher Scientific	Cat# A11029 RRID: AB_2534088
Alexa Fluor 488 conjugated goat anti-rabit IgG	Thermo Fisher Scientific	Cat# A11034 RRID: AB_2758380
Alexa Fluor Plus 594 conjugated goat anti-mouse IgG	Thermo Fisher Scientific	Cat# A32742 RRID: AB_2762825

Alexa Fluor 594 conjugated donkey anti-goat IgG	Thermo Fisher Scientific	Cat# A11058<br>RRID: AB_2758385
Alexa Fluor 488 conjugated goat anti-rat	Thermo Fisher Scientific	Cat# A-11006; RRID: AB_2534074
Alexa Fluor Plus 555 conjugated goat anti-rabbit	Thermo Fisher Scientific	Cat# A32732; RRID: AB_2633281
Alexa Fluor 647 conjugated goat anti-rabbit	Thermo Fisher Scientific	Cat# A-21244; RRID: AB_2535812
Alexa Fluor 555 conjugated goat anti-guinea pig	Thermo Fisher Scientific	Cat# A-21435; RRID: AB_2535856
Alexa Fluor 647 conjugated goat anti-guinea pig	Thermo Fisher Scientific	Cat# A-21450; RRID: AB_2735091
Alexa Fluor 647 conjugated goat anti-mouse IgG1	Thermo Fisher Scientific	Cat# A-21240; RRID: AB_2535809
Alexa Fluor 488 conjugated goat anti-mouse IgG2a	Thermo Fisher Scientific	Cat# A-21131; RRID: AB_2535771
**Bacterial and Virus Strains**		
Stellar competent cells	Takara	Cat# 636763
NEB® Stable Competent E. coli (High Efficiency)	New England Biolabs	Cat# C3040H
NEB® 5-alpha Competent E. coli (High Efficiency)	New England Biolabs	Cat# C2987H
AAV PHP.S encapsulated vectors	This paper	N/A
AAV PHP.eB encapsulated vectors	This paper	N/A
**Chemicals, Peptides, and Recombinant Proteins**		
Biotin tyramide	Perkin Elmer	Cat# NEL749A001KT
Neurobasal Medium	Gibco	Cat# 21103-049
B-27 supplement	Gibco	Cat# 17504-044
GlutaMax-I	Gibco	Cat# 35050-061
Penicillin-Streptomycin	Gibco	Cat# 15140122
DMEM, high glucose, no glutamine	Gibco	Cat# 11960-044
Fetal bovine serum	Cytiva	Cat# SH30109.03
0.5% Trypsin-EDTA	Gibco	Cat# 15400054
2.5% trypsin	Gibco	Cat# 15090046
Hank's buffered salt solution (HBSS)	Gibco	Cat# 14175-095
Poly-D-lysine	Gibco	Cat# A3890401
Poly-L-lysine	Sigma	Cat# P4832-50ML
Laminin	Gibco	Cat# 23017015
Phosphate buffer saline (PBS)	Corning	Cat# 21-031-CV
Opti-MEM	Thermo Fisher Scientific	Cat# 31985070
Hoechst 33258	Thermo Fisher Scientific	Cat# H3569<br>RRID: AB_2651133
Polyethylenimine (PEI)	Polysciences	Cat# 24765-1
Normal goat serum	Gibco	Cat# 16210-072
Gelatin from bovine skin	Sigma	G9382-100G
FastGreen	Sigma	Cat# F7258
Streptavidin Mag Sepharose	GE Healthcare	Cat# 28-9857-38
Vectashield plus anti-fade mounting media	Vector labs	Cat# H-1900
Sequencing Grade Trypsin	Promega	V5111
Tamoxifen	Sigma-Aldrich	Cat# T5648; CAS: 10540-29-1

Fluoromount-G	Southern Biotech	0100-01
**Critical Commercial Assays**		
Q5 High-Fidelity DNA Polymerase	New England Biolabs	Cat# M0491S
CloneAmp HiFi PCR Premix	Takara	Cat# 639298
NucleoSpin Plasmid Transfection-grade	Takara	Cat# 740490.250
In-Fusion Snap Assembly Master Mix	Takara	Cat# 638947
Gel purification	Cytiva	28903470
Amicon Ultra-15 Centrifugal Filter Unit 100 kDa	Millipore	Cat# UFC910008
C18 ZipTips	Millipore	Cat# ZTC18S096
BamHI restriction enzyme	New England Biolabs	Cat# R3136T
EcoRI restriction enzyme	New England Biolabs	Cat# R3101T
NotI restriction enzyme	New England Biolabs	Cat# R3189L
XhoI restriction enzyme	New England Biolabs	Cat# R0146M
**Experimental models: Cell Lines**		
HEK293T cells	Provided from Baylor College of Medicine Neuroconnectivity Core	N/A
**Experimental models: Organisms/ Strains**		
Rat: Sprague-Dawley rat embryos	Charles River Laboratories	SAS-SD
Mouse: C57BL/6	Baylor College of Medicine Center for Comparative Medicine	N/A
Mouse: CFW (Swiss Webster)	Charles River	Cat# CRL:24; RRID: IMSR_CRL:24
Mouse: Nkx2-1$^{tm1.1(Cre/ERT2)Zjh}$/J	Gift from Z.J. Huang; (Taniguchi et al., 2013)	JAX: 014552; RRID: IMSR_JAX:014552
Mouse: B6;129S6-Gt(Rosa)26Sor$^{tm9(CAG-tdTomato)Hze}$/J	Gift from Z.J. Huang; (Taniguchi et al., 2013)	JAX: 007905; RRID: IMSR_JAX:007905
Mouse: Igs2tm1.1(CAG-cas9*)Mmw/J (also known as H11Cas9 mice)	The Jackson Laboratory	Cat# JAX: 027650 RRID: IMSR_JAX:027650
Mouse: Cntn1 knockout	Boyle et al., 2001	Cat# JAX: q034216, RRID: IMSR_JAX:034216
Mouse: ICR	Baylor College of Medicine Center for Comparative Medicine	N/A
**Recombinant DNA plasmids**		
PX551 (AAV-SpCas9)	Gift of Dr. Feng Zhang	Addgene #60957
PX552 (pAAV-U6sgRNA(SapI)_hSyn-GFP-KASH-bGH)	Gift of Dr. Feng Zhang	Addgene #60958
pCAG-1BPNLS-Cas9-1BPNLS	Gift of Dr. Juan Belmonte	Addgene #87108
pCAG_smFP HA	Gift of Dr. Loren Looger	Addgene #59759
pCAG_smFP V5	Gift of Dr. Loren Looger	Addgene #59758
pMJ114	Gift of Dr. Jonathan Weissman	Addgene #85995
pMJ117	Gift of Dr. Jonathan Weissman	Addgene #85997
pMJ179	Gift of Dr. Jonathan Weissman	Addgene #85996
pUCmini-iCAP-PHP.S	Gift of Dr. Viviana Gradinaru	Addgene #103002
pUCmini-iCAP-PHP.eB	Gift of Dr. Viviana Gradinaru	Addgene #103005
pHelper	Agilent Technologies	Cat #240071

Knock-in sgRNA and donor plasmid	This paper	N/A
pAAV-3x-gRNA-smFP	This paper	N/A
rat contactin-myc	Gift of Dr. Elior Peles	N/A
pAAV-hSyn-EGFP	Gift of Dr. Bryan Roth	Addgene # 5046
pcDNA3	Invitrogen	N/A
hSYNp-Cntn1-Myc full-length	This paper	N/A
hSYNp-Cntn1-Myc truncates	This paper	N/A
**Software and Algorithms**		
Fiji	NIH	https://fiji.sc/
Zeiss Zen (Blue Edition) Imaging Software	Zeiss	http://www.zeiss.com/microscopy/en_us/products/microscope-software/zen.html#introduction; RRID: SCR_013672
NIS Elements Imaging Software	Nikon	https://www.microscope.healthcare.nikon.com/products/software/nis-elements
CRISPOR	Haeussler et al., 2016	http://crispor.tefor.net/crispor.py
Adobe Photoshop (version 23.5.1)	Adobe	https://www.adobe.com/products/photoshop.html
Adobe Illustrator (version 26.5)	Adobe	https://www.adobe.com/products/illustrator.html
GraphPad Prism 9	GraphPad	https://www.graphpad.com/
MATLAB	Mathworks	https://www.mathworks.com/products/matlab.html
Python	Python Software Foundation	https://www.python.org/
Script for calculation of extracellular tyrosines	Rasband lab	https://github.com/jrasband/extracellular-tyrosines
BioRender	BioRender	https://biorender.com/
ShinyGO v0.77	ShinyGO	http://bioinformatics.sdstate.edu/go/
**Other**		
Axioimager Z2 with Apotome	Zeiss	
Ni2 upright fluorescence microscope	Nikon	
Microtome HM450	Thermo Scientific	
Cryostat CryoStar NX70	Thermo Scientific	
QExactive Plus	Thermo Scientific	
NanoAcquity Ultra Performance UPLC system	Waters	
15-cm EasySpray C18 column	Thermo Scientific	
