# Supplementary material for: Antibody-directed extracellular proximity biotinylation reveals that Contactin-1 regulates axo-axonic innervation of axon initial segments
Source: Nat Commun. 2023 Oct 26;14:6797. doi: 10.1038/s41467-023-42273-8 (PMC10603070; doi:10.1038/s41467-023-42273-8)
Supplement: Supplementary file 3 — Description of Additional Supplementary Files [file 41467_2023_42273_MOESM3_ESM.pdf]

Description of Additional Supplementary Files

File Name: Supplementary Data 1
Description: Comparison of Nfasc-BAR with NrCAM-BAR.

File Name: Supplementary Data 2
Description: Proteomics results from Nfasc-BAR across development.

File Name: Supplementary Data 3
Description: fold-change and statistical comparison across development. Data were used to generate volcano plots.

File Name: Supplementary Data 4
Description: gRNA sequences used in this study.
