# Supplementary material for: Antibody-directed extracellular proximity biotinylation reveals that Contactin-1 regulates axo-axonic innervation of axon initial segments
Source: Nat Commun. 2023 Oct 26;14:6797. doi: 10.1038/s41467-023-42273-8 (PMC10603070; doi:10.1038/s41467-023-42273-8)
Supplement: Supplementary file 8 — Reporting Summary [file 41467_2023_42273_MOESM8_ESM.pdf]

nature portfolio

Corresponding author(s): Rasband, MN

Last updated by author(s): Jul 7, 2023

Reporting Summary

Nature Portfolio wishes to improve the reproducibility of the work that we publish. This form provides structure for consistency and transparency in reporting. For further information on Nature Portfolio policies, see our Editorial Policies and the Editorial Policy Checklist.

Statistics

For all statistical analyses, confirm that the following items are present in the figure legend, table legend, main text, or Methods section.

n/a	Confirmed	
☐	☒	The exact sample size (*n*) for each experimental group/condition, given as a discrete number and unit of measurement
☐	☒	A statement on whether measurements were taken from distinct samples or whether the same sample was measured repeatedly
☐	☒	The statistical test(s) used AND whether they are one- or two-sided<br>*Only common tests should be described solely by name; describe more complex techniques in the Methods section.*
☒	☐	A description of all covariates tested
☒	☐	A description of any assumptions or corrections, such as tests of normality and adjustment for multiple comparisons
☐	☒	A full description of the statistical parameters including central tendency (e.g. means) or other basic estimates (e.g. regression coefficient) AND variation (e.g. standard deviation) or associated estimates of uncertainty (e.g. confidence intervals)
☐	☒	For null hypothesis testing, the test statistic (e.g. *F*, *t*, *r*) with confidence intervals, effect sizes, degrees of freedom and *P* value noted<br>*Give P values as exact values whenever suitable.*
☒	☐	For Bayesian analysis, information on the choice of priors and Markov chain Monte Carlo settings
☒	☐	For hierarchical and complex designs, identification of the appropriate level for tests and full reporting of outcomes
☒	☐	Estimates of effect sizes (e.g. Cohen's *d*, Pearson's *r*), indicating how they were calculated

Our web collection on statistics for biologists contains articles on many of the points above.

Software and code

Policy information about availability of computer code

Data collection	For microscopy the following software was used:<br>1. Zeiss Zen (Blue Edition) Imaging Software Zeiss http://www.zeiss.com/microscopy/en_us/products/microscope-software/zen.html#introduction; RRID: SCR_013672<br>2. NIS Elements Imaging Software Nikon https://www.microscope.healthcare.nikon.com/products/software/nis-elements
Data analysis	For data analysis the following software tools were used or generated:<br>1. Fiji NIH (version 2.14.0/1.54d) https://fiji.sc/<br>2. CRISPOR (version 5.01) Haeussler et al., 2016 http://crispor.tefor.net/crispor.py<br>3. Adobe Photoshop (version 23.5.1) Adobe https://www.adobe.com/products/photoshop.html<br>4. Adobe Illustrator (version 26.5) Adobe https://www.adobe.com/products/illustrator.html<br>5. GraphPad Prism 9 GraphPad https://www.graphpad.com/<br>6. MATLAB Mathworks https://www.mathworks.com/products/matlab.html<br>7. Python Python Software Foundation https://www.python.org/<br>8. Script for calculation of extracellular tyrosines Rasband lab https://github.com/jrasband/extracellular-tyrosines<br>9. BioRender BioRender https://biorender.com/<br>10. ShinyGO v0.77 ShinyGO http://bioinformatics.sdstate.edu/go/

For manuscripts utilizing custom algorithms or software that are central to the research but not yet described in published literature, software must be made available to editors and reviewers. We strongly encourage code deposition in a community repository (e.g. GitHub). See the Nature Portfolio guidelines for submitting code & software for further information.

Data

Policy information about availability of data

All manuscripts must include a data availability statement. This statement should provide the following information, where applicable:

- Accession codes, unique identifiers, or web links for publicly available datasets
- A description of any restrictions on data availability
- For clinical datasets or third party data, please ensure that the statement adheres to our policy

All data generated or analyzed during this study are included in this published article. All proteomics data have been deposited to the ProteomeXchange Consortium via the PRIDE partner repository with the identifier PXD043805. Supplemental material including all PSMs are including in the supplemental tables. The source data underlying Figs. 4c-d, 6d, h, k, 7d, f, i, 8c, 9d, f, h, S1b, S6a-d, S7c-d, S8a-c are provided in the Source Data file.

Research involving human participants, their data, or biological material

Policy information about studies with human participants or human data. See also policy information about sex, gender (identity/presentation), and sexual orientation and race, ethnicity and racism.

Reporting on sex and gender	N/A
Reporting on race, ethnicity, or other socially relevant groupings	N/A
Population characteristics	N/A
Recruitment	N/A
Ethics oversight	N/A

Note that full information on the approval of the study protocol must also be provided in the manuscript.

Field-specific reporting

Please select the one below that is the best fit for your research. If you are not sure, read the appropriate sections before making your selection.

☒ Life sciences ☐ Behavioural & social sciences ☐ Ecological, evolutionary & environmental sciences

For a reference copy of the document with all sections, see nature.com/documents/nr-reporting-summary-flat.pdf

Life sciences study design

All studies must disclose on these points even when the disclosure is negative.

Sample size	Sample size N were independent experiments or animals. In some experiments n were the number of cells counted in a given experiment. No statistical tests were used to predetermine sample sizes as the research is exploratory and no prior experiment could be referred to. In some instances, sample sizes were determined based on prior publications performing similar analyses (e.g. Tai et al., Neuron 2019).
Data exclusions	no data were excluded from the analyses.
Replication	Both biological and technical replicates were performed. The number of independent experiments used to replicate studies is reported in the methods or figure legends. Mouse models, consistent biological replicates, materials and quantities used, and methodology were all provided in full transparency for ease of replication by others.
Randomization	There was no randomization as all conditions were included in each experiment
Blinding	In all experiments using sgRNA knockout of Cntn1 or other genes, investigators were blinded to treatment (e.g. data presented in Figs. 6, S6, 7, S7, 9, and S8). It was not possible to be blinded to the genotype of Cntn1 KO mice (Fig. 8 since changes to Pinceau structure were so obvious).

Reporting for specific materials, systems and methods

We require information from authors about some types of materials, experimental systems and methods used in many studies. Here, indicate whether each material, system or method listed is relevant to your study. If you are not sure if a list item applies to your research, read the appropriate section before selecting a response.

Materials & experimental systems

n/a	Involved in the study
☐	☒ Antibodies
☐	☒ Eukaryotic cell lines
☒	☐ Palaeontology and archaeology
☐	☒ Animals and other organisms
☒	☐ Clinical data
☒	☐ Dual use research of concern
☒	☐ Plants

Methods

n/a	Involved in the study
☒	☐ ChIP-seq
☒	☐ Flow cytometry
☒	☐ MRI-based neuroimaging

Antibodies

Antibodies used

Mouse anti-AnkG (IgG2a) NeuroMab Cat# 75-146; RRID: AB_10673030
Mouse anti-Gephyrin (IgG1) Synaptic Systems Cat# 147011; RRID: AB_887717
Guinea pig anti-VGAT Synaptic Systems Cat# 131004; RRID: AB_887873
Guinea pig anti-Brevican Gift of Dr. Constanze Seidenbecher N/A
Rabbit monoclonal anti-HA Cell Signaling Technology Cat# 3724; RRID: AB_1549585
Mouse anti-Myc MBL International Corporation Cat# M192
PRID: AB_11160947
Mouse anti-PSD95 Antibodies Incorporated Cat# 75-028
PRID: AB_2292909
Mouse anti-Tenascin-R R&D Systems Cat# MAB1624
RRID: AB_2207001
Mouse anti-Tuj1 BioLegend Cat# 801202
RRID: AB_10063408
Mouse anti-V5 Invitrogen Cat# R960CUS
RRID: AB_159298
Rabbit anti-βIV-Spectrin Rasband lab RRID: AB_2315634
Rabbit anti-Kv1.2 Gift of Dr. James Trimmer RRID: 2756300
Rabbit anti-NrCAM Abcam Cat# ab24344
RRID: AB_448024
Chicken anti-MAP2 Encor Cat# CPCA-MAP2
Chicken anti-Neurofascin R&D Systems Cat# AF3235
RRID: AB_10890736
Goat anti-Cntn1 R&D Systems Cat# AF904
RRID: AB_2292070
Rat anti-HA Millipore Sigma Cat# 11867423001
RRID: AB_390918
HRP-conjugated goat anti-chicken IgY Aves Labs Cat# H-1004
RRID: AB_2313517
HRP-conjugated goat anti-rabbit IgG Jackson ImmunoResearch Labs Cat# 111-035-003
PRID: AB_2313567
Alexa Fluor 594 conjugated streptavidin Thermo Fisher Scientific Cat# S11227
Aminomethylcoumarin (AMCA) conjugated goat anti-chicken IgY Jackson ImmunoResearch Labs Cat# 103-155-155
RRID: AB_2337385
Aminomethylcoumarin (AMCA) conjugated goat anti-rat IgG Thermo Fisher Scientific Cat# A21093
PRID: AB_2535748
Alexa Fluor 488 conjugated goat anti-chicken IgY Jackson ImmunoResearch Labs Cat# 103-545-155
RRID: AB_2337390
Alexa Fluor 488 conjugated goat anti-mouse IgG Thermo Fisher Scientific Cat# A11029
RRID: AB_2534088
Alexa Fluor 488 conjugated goat anti-rabit IgG Thermo Fisher Scientific Cat# A11034
RRID: AB_2758380
Alexa Fluor Plus 594 conjugated goat anti-mouse IgG Thermo Fisher Scientific Cat# A32742
RRID: AB_2762825
Alexa Fluor 594 conjugated donkey anti-goat IgG Thermo Fisher Scientific Cat# A11058
RRID: AB_2758385
Alexa Fluor 488 conjugated goat anti-rat Thermo Fisher Scientific Cat# A-11006; RRID:
AB_2534074
Alexa Fluor Plus 555 conjugated goat anti-rabbit Thermo Fisher Scientific Cat# A32732; RRID: AB_2633281
Alexa Fluor 647 conjugated goat anti-rabbit Thermo Fisher Scientific Cat# A-21244; RRID: AB_2535812
Alexa Fluor 555 conjugated goat anti-guinea pig Thermo Fisher Scientific Cat# A-21435; RRID: AB_2535856
Alexa Fluor 647 conjugated goat anti-guinea pig Thermo Fisher Scientific Cat# A-21450; RRID:
AB_2735091
Alexa Fluor 647 conjugated goat anti-mouse IgG1 Thermo Fisher Scientific Cat# A-21240; RRID:
AB_2535809
Alexa Fluor 488 conjugated goat anti-mouse IgG2a Thermo Fisher Scientific Cat# A-21131; RRID: AB_2535771

Validation

Antibodies against AnkG are knockout validated. See Chang et al., Nat Neurosci 17:1673-81 (2014)

Antibodies against Neurofascin are knockout validated. See Amor et al., eLIFE 6:e21392 (2017).

Antibodies against Gephyrin are knockout validated. See Feng et al., Science 282:1321-4 (1998)

Antibodies against Brevican are knockout validated. see Susuki et al., Neuron 2013.

Antibodies against PSD95 are knockout validated (https://neuromab.ucdavis.edu/datasheet/K28_43.pdf)

Antibodies against βIV-Spectrin are knockout validated, see Yang et al., J Neurosci 24:7230-7240 (2004).

Rabbit anti-Kv1.2 Gift of Dr. James Trimmer RRID: 2756300

Map2, Tuj1, Tenascin-R, and VGAT antibodies were validated by immunolabeling and/or immunoblot consistent with their data sheets: MAP2 (http://encorbio.com/products/cpca-map2/), Tuj1 (https://www.biolegend.com/en-us/products/purified-anti-tubulin-beta-3-tubb3-antibody-11580), Tnr (https://www.rndsystems.com/products/mouse-rat-tenascin-r-antibody-619_mab1624#product-details), VGAT (https://sysy.com/product/131004).

Antibodies against Cntn1 and NrCAM were validated in this paper using sgRNA knockout. See Figures 6b-d and 7e-f.

Antibodies against HA, V5, myc, RFP, and GFP were validated by transfecting cells with plasmids expressing proteins fused to these tags. Both immunostaining and immunoblots were used to validate the antibodies.

All secondary antibodies were validated by testing for binding against their target primary antibody species without cross-reactivity against other species.

Eukaryotic cell lines

Policy information about cell lines and Sex and Gender in Research

Cell line source(s): HEK293T cells were obtained from ATCC.

Authentication: Cell lines were not authenticated.

Mycoplasma contamination: cells lines were not tested for mycoplasma contamination.

Commonly misidentified lines (See ICLAC register): *Name any commonly misidentified cell lines used in the study and provide a rationale for their use.*

Animals and other research organisms

Policy information about studies involving animals; ARRIVE guidelines recommended for reporting animal research, and Sex and Gender in Research

Laboratory animals

Rat: Sprague-Dawley rat embryos Charles River Laboratories SAS-SD
Mouse: C57BL/6 Baylor College of Medicine Center for Comparative Medicine N/A
Mouse: CFW (Swiss Webster) Charles River Cat# CRL:24; RRID: IMSR_CRL:24
Mouse: Nkx2-1tm1.1(Cre/ERT2)Zjh/J Gift from Z.J. Huang;
(Taniguchi et al., 2013)
JAX: 014552; RRID: IMSR_JAX:014552
Mouse: B6;129S6-Gt(Rosa)26Sortm9(CAG-tdTomato)Hze/J Gift from Z.J. Huang;
(Taniguchi et al., 2013)
JAX: 007905; RRID: IMSR_JAX:007905
Mouse: Igs2tm1.1(CAG-cas9*)Mmw/J
(also known as H11Cas9 mice) The Jackson Laboratory Cat# JAX: 027650
RRID: IMSR_JAX:027650
Mouse: Cntn1 knockout Boyle et al., 2001 Cat# JAX: q034216, RRID: IMSR_JAX:034216
Mouse: ICR Baylor College of Medicine Center for Comparative Medicine N/A

Ages of mice used: Fig. 6g,h, 13 weeks; Fig. 6j,k P0; Fig. 8, P17; Fig. 9, P17.
Ages of rats to generate primary neurons: adult rats and E18.5 embryos.

mice were maintained in a facility with a 12h light/12 h dark cycle. Room temperatures were ~22 C with 40-60% humidity.

Wild animals: no wild animals were used in the study

Reporting on sex: There was no consideration of sex for primary neuronal cultures. Cultures were mixed. For Cntn1 KO mice sex was also not considered since it was so difficult to obtain KO mice (they usually died prior to our ability to analyze them), therefore we used whatever sex was available. We also did not consider sex in the in utero electroporation experiments.

Field-collected samples	no field collected samples were used in the study
Ethics oversight	All animal studies were approved by Baylor College of Medicines IACUC and performed in accordance with the NIH guide for the humane care and use of animals. Protocol number AN-4634. Some experiments were performed at Cold Spring Harbor labs using animal protocol number 22-19-16-13-10-06-7. Some experiments were performed at the Weizmann Institute of Science using animal protocol number 00530121.

Note that full information on the approval of the study protocol must also be provided in the manuscript.
